# Supplementary material for: Long-Term Oncological Outcomes of Granulocyte Colony-Stimulating Factor (G-CSF) Treatment in Gastrointestinal Cancers: A Systematic Review and Meta-Analysis
Source: Cancers (Basel). 2025 Apr 14;17(8):1313. doi: 10.3390/cancers17081313 (PMC12026166; doi:10.3390/cancers17081313)
Supplement: Supplementary file 1 [file cancers-17-01313-s001.zip › cancers-3520719-supplementary.pdf]

Supplementary Table S1 – Search Strategy and Keywords.

Participants (P)	AND	Intervention (I)	AND	Comparator (C)	AND	Outcome (O)
Upper gastrointestinal cancers<br>OR<br>Esophageal cancer<br>OR<br>Esophageal neoplasia<br>OR<br>Oesophageal cancer<br>OR<br>Gastroesophageal junction cancer<br>OR<br>gastric cancer<br>OR<br>gastric neoplasm<br>OR<br>gastric neoplasia<br>OR<br>Small bowel cancer<br>OR<br>Small intestine cancer<br>OR<br>Pancreatic cancer<br>OR<br>PDAC<br>OR<br>pancreatic ductal adenocarcinoma<br>OR<br>pancreatic neoplasms<br>OR<br>Pancreatic neoplasia<br>OR<br>pancreas cancer<br>OR<br>liver cancers<br>OR<br>hepatocellular carcinoma<br>OR<br>cholangiocarcinoma<br>OR<br>gallbladder cancer<br>OR<br>Gallbladder carcinoma<br>OR<br>Lower gastrointestinal cancer<br>OR<br>colon cancer<br>OR<br>colorectal cancer<br>OR<br>colorectal neoplasms<br>OR<br>colorectal neoplasia<br>OR<br>rectal cancer		Granulocyte colony stimulating factor<br>OR<br>Granulocyte colony-stimulating factor<br>OR<br>G-CSF<br>OR<br>GCSF<br>OR<br>Filgrastim<br>OR<br>Pegfilgrastim<br>OR<br>Lipegfilgrastim<br>OR<br>Lenograstim		None		Recurrence<br>OR<br>Cancer recurrence<br>OR<br>Cancer prognosis<br>OR<br>Prognosis<br>OR<br>Overall survival<br>OR<br>Cancer-specific survival<br>OR<br>Disease-free survival<br>OR<br>Progression-free survival<br>OR<br>Relapse<br>OR<br>Adverse events<br>OR<br>Event-free survival<br>OR<br>MDSC<br>OR<br>MDSCs<br>OR<br>myeloid-derived suppressor cells<br>OR<br>polymorphonuclear MDSCs<br>OR<br>PMN-MDSC<br>OR<br>monocytic MDSC<br>OR<br>M-MDSCs

Supplementary Table S2 – Search string utilized in Embase

1.	upper gastrointestinal cancer.mp.
2.	esophagus cancer/
3.	esophageal adenocarcinoma/ or esophagus tumor/ or esophagus carcinoma/
4.	Oesophageal cancer.mp.
5.	stomach cancer/ or Gastroesophageal junction cancer.mp.
6.	Gastric cancer.mp.
7.	stomach tumor/ or gastric neoplasm.mp.
8.	gastric neoplasia.mp. or stomach adenocarcinoma/
9.	small intestine cancer/ or small bowel cancer.mp. or intestine cancer/
10.	pancreatic cancer.mp. or pancreas cancer/
11.	pancreas adenocarcinoma/ or PDAC.mp.
12.	pancreatic ductal adenocarcinoma.mp. or pancreatic ductal carcinoma/
13.	pancreatic neoplasm.mp. or pancreas tumor/
14.	pancreatic neoplasia.mp.
15.	liver cancer/
16.	hepatocellular carcinoma.mp. or liver cell carcinoma/
17.	cholangiocarcinoma.mp. or bile duct carcinoma/
18.	gallbladder cancer/
19.	gallbladder carcinoma/
20.	digestive system cancer/ or lower gastrointestinal cancer.mp.
21.	colon cancer/
22.	colon tumor/ or rectum carcinoma/ or colorectal cancer/ or colon carcinoma/ or rectum cancer/ or rectum tumor/
23.	colorectal neoplasm.mp. or colorectal tumor/
24.	colorectal neoplasia.mp.
25.	rectal cancer.mp.
26.	1 or 2 or 3 or 4 or 5 or 6 or 7 or 8 or 9 or 10 or 11 or 12 or 13 or 14 or 15 or 16 or 17 or 18 or 19 or 20 or 21 or 22 or 23 or 24 or 25

27. granulocyte colony stimulating factor/
28. granulocyte colony-stimulating factor.mp.
29. G-CSF.mp.
30. recombinant granulocyte colony stimulating factor/ or GCSF.mp.
31. filgrastim/
32. pegfilgrastim/
33. lipegfilgrastim/
34. lenograstim/
35. 27 or 28 or 29 or 30 or 31 or 32 or 33 or 34
36. recurrence.mp. or recurrent disease/
37. cancer recurrence/
38. prognosis/ or cancer prognosis/
39. overall survival/
40. cancer specific survival/
41. progression free survival/
42. relapse/
43. adverse event/
44. event free survival/
45. suppressor cell/ or MDSC.mp. or myeloid-derived suppressor cell/
46. MDSCs.mp.
47. polymorphonuclear MDSC.mp.
48. PMN MDSC.mp.
49. M-MDSC.mp.
50. monocytic MDSC.mp.
51. 36 or 37 or 38 or 39 or 40 or 41 or 42 or 43 or 44 or 45 or 46 or 47 or 48 or 49 or 50
52. 26 and 35 and 51

Hits = 985 studies.

Supplementary Materials S1 – Eligibility Criteria

<u>**P**articipants:</u>

Patients with gastrointestinal cancer, including esophageal, gastroesophageal junction, gastric, hepatocellular, intra- and extrahepatic bile duct, pancreatic and colorectal cancers, treated with G-CSF alongside myelosuppressive/myeloablative chemotherapy. There are no restrictions regarding IUCC stage or prior surgical resection.

<u>**I**ntervention/exposure:</u>

G-CSF treatment.

<u>**C**omparison:</u>

None.

<u>**O**utcome:</u>

Main outcomes are overall survival, cancer-specific survival, disease-free survival, progression-free survival, adverse effects and circulating MDSC levels.

<u>Study design:</u>

No restrictions regarding study design.

<u>Criteria for exclusion:</u>

The following criteria resulted in exclusion from the study:

- Reviews;
- Editorial letters;
- Abstracts or conference posters;
- Commentaries;
- Case reports;
- Animal studies;
- Studies in foreign languages that cannot be translated.

Supplementary Table S3 - The Newcastle–Ottawa Quality Assessment Scale (NOS), used to assess risk of bias in included cohort studies.

	Exposed	Non-Exposed	Ascertainment	Outcome	Comparability	Assessment	Follow-up	Adequacy	Total
**Pitot H.C. et al. 2000**	★	★	★	-	★	★	★	★	7
**Amadio A. et al. 2014**	★	★	★	-	★★	★	★	★	8
**Yamao K. et al. 2019**	★	★	★	-	★★	★	★	★	8
**Jung J.H. et al. 2020**	★	★	★	-	★	★	★	★	7
**Canton C. et al. 2022**	★	★	★	-	★	-	★	★	6
**Okamoto K. et al. 2022**	★	★	★	-	★★	★	★	★	8

Supplementary Table S4 - The Cochrane Risk of Bias Tool for Randomized Controlled Trials.

	Random sequence generation	Allocation concealment	Selective reporting	Other bias	Blinding (participants and personnel)	Blinding (outcome assessment)	Incomplete outcome data	AHRQ Standards assesment
**Wadler S. et al. 2002**	Low risk	Unclear	Unclear	Low risk	Low risk	Low risk	Low risk	Fair quality
**Cascinu S. et al 2007**	Unclear	Unclear	Low risk	Low risk	Low risk	Low risk	Low risk	Good quality
**Hecht J.R. et al. 2010**	Low risk	Unclear	Low risk	Low risk	Low risk	Low risk	Low risk	Good quality
**Lasalvia-Prisco E. et al. 2012**	Low risk	Unclear	Low risk	Low risk	Unclear	Unclear	Low risk	Poor quality
**Shah M.A. et al. 2015**	Low risk	Unclear	Low risk	Low risk	Low risk	Unclear	Low risk	Good quality
**Chen J. et al. 2017**	Low risk	Unclear	Low risk	Low risk	Unclear	Low risk	Low risk	Poor quality
**Pinter T. et al. 2017**	Low risk	Low risk	Low risk	Low risk	Low risk	Low risk	Low risk	Good quality

Supplementary Table S5 – Studies included in the review but excluded from data synthesis.

Study	Cancer
Rowinsky E.K. et al. 1998 [42]	Colorectal
Saltz L.B. et al. 1997 [43]	Colorectal
Ajani J.A. et al. 1994 [44]	Esophageal
Donlon N.E. et al. 2021 [45]	Esophageal
Ohtsu A. et al. 1999 [46]	Esophageal
Sharma A. et al. 2021 [47]	Gallbladder
Catenacci D.V.T. et al. 2020 [48]	Gastric
Chiesa M.D. et al. 2011 [49]	Gastric
DiLauro L. et al. 2005 [50]	Gastric
Felici A. et al. 2006 [51]	Gastric
Hejna M. et al. 2008 [52]	Gastric
Makatsoris T. et al. 2007 [53]	Gastric
Mavroudis D. et al. 2000 [54]	Gastric
Shin S.J. et al. 2008 [55]	Gastric
Bojic M. et al. 2011 [56]	Gastric
Dirican A. et al. 2013 [57]	Gastric
Liguigli W. et al. 2017 [58]	Gastric
Ozdemir N. et al. 2014 [59]	Gastric
Tomasello G. et al. 2010 [60]	Gastric
Tomasello G. et al. 2014 [61]	Gastric
Xiao J. et al. 2015 [62]	Gastric
Tomasello G. et al. 2017 [63]	Gastric
Li B. et al. 2003 [64]	Hepatic
Bamias A. et al. 2004 [65]	Mixed GI
Androulakis N. et al. 1999 [66]	Pancreatic
Lakatos G. et al. 2017 [67]	Pancreatic
Marsh R.W. et al. 2018 [68]	Pancreatic
Sasaki M. et al. 2021 [69]	Pancreatic
Scheithauer W. et al. 1999 [70]	Pancreatic
Ghorani E. et al. 2015 [71]	Pancreatic
Sijde F. et al. 2022 [72]	Pancreatic
Caron B. et al. 2021 [73]	Pancreatic
Macdonald J.S. et al. 2000 [74]	Pancreatic
Mahaseth H. et al. 2013 [75]	Pancreatic
Stathopoulos G.P. et al. 2001 [76]	Pancreatic
Tsubamoto H. et al. 2015 [77]	Pancreatic
Stein S.M. et al. 2016 [78]	Pancreatic

Supplementary Figure S1: Forrest plot on subgroup analysis in regard to location of cancer in all studies reporting on OS.

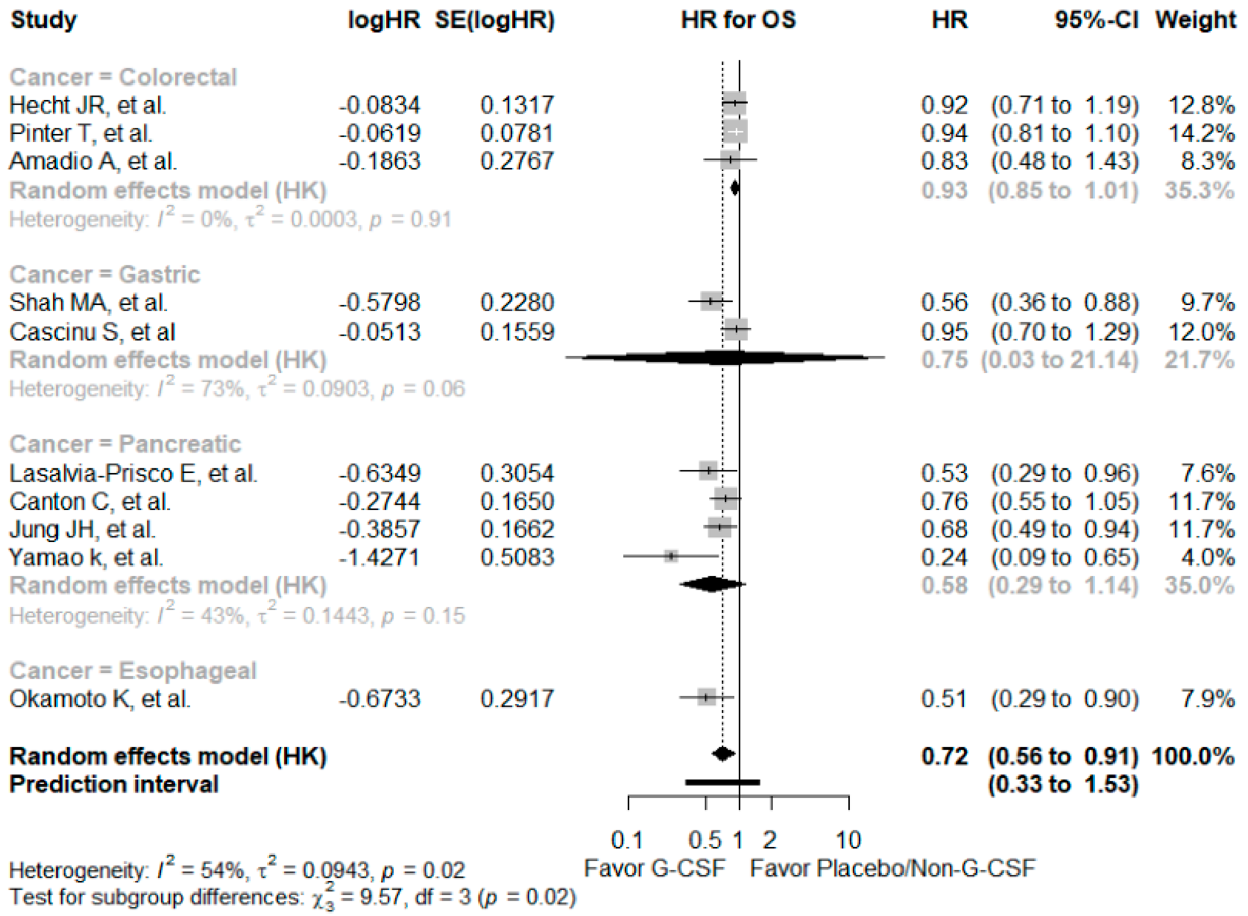

Supplementary Figure S2: Forrest plot on subgroup analysis in regard to location of cancer in all studies reporting on PFS.

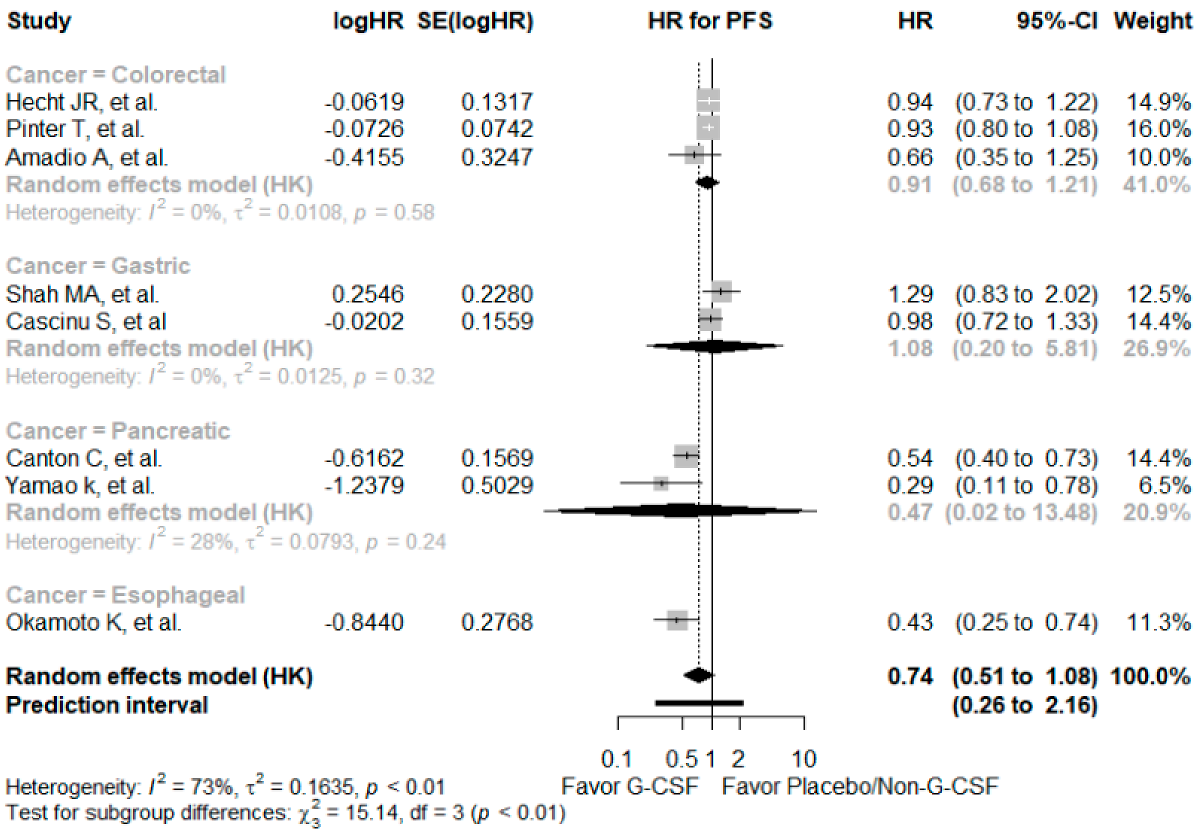

Supplementary Figure S3: Forrest plot on subgroup analysis in regard to location of cancer in all studies reporting on AE.

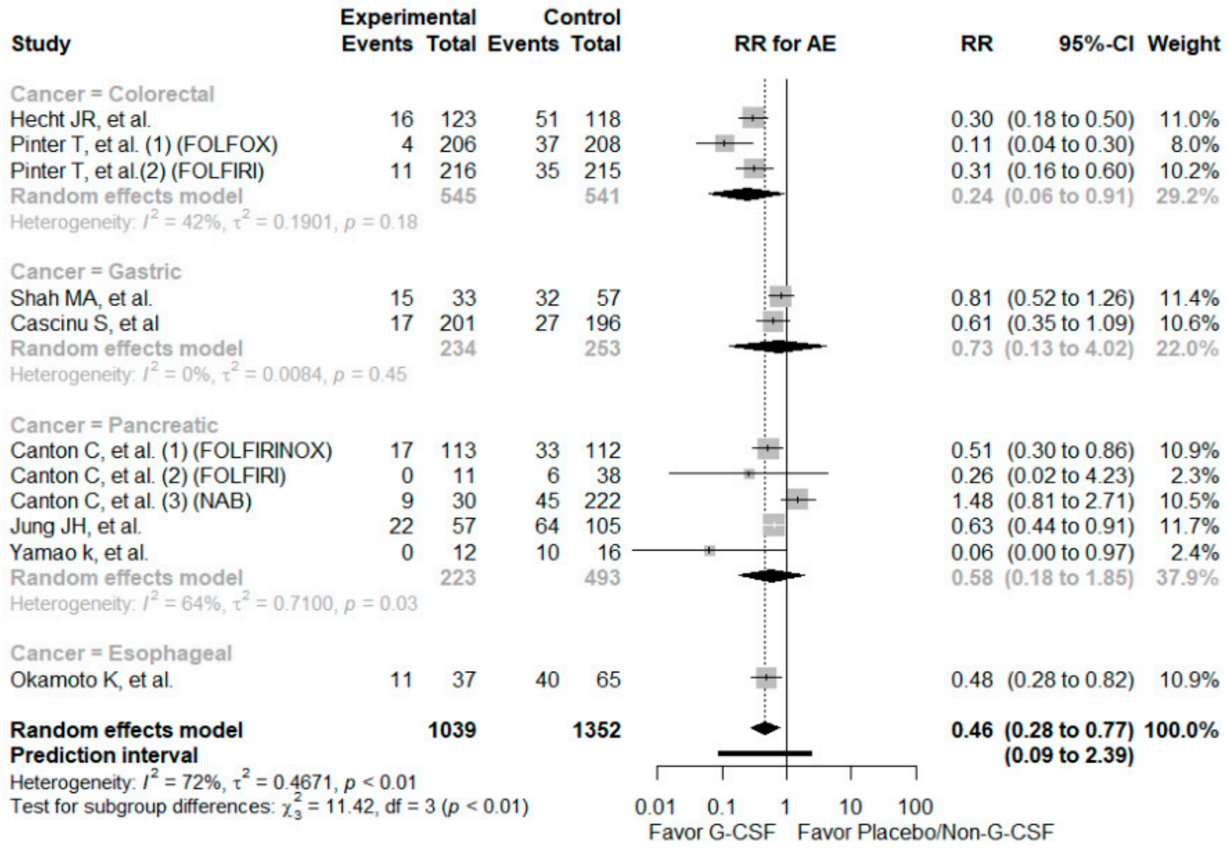

Supplementary Materials S2—GRADE Approach in Detail

Overall Survival

Risk of bias

Risk of bias was assessed using the Newcastle–Ottawa Scale in retrospective cohort trials and the Risk of Bias (Rob) tool was utilized in the RCTs. Only one of the included studies (Lasalvia-Prisco et al.) was of poor quality. The risk of bias was thus low, and the evidence was therefore not downgraded.

Inconsistency of results

The heterogeneity of the results obtained from the time-to-event meta-analysis on OS revealed a I^2 value of 54 % with and without subgroup analysis. The evidence was downgraded for inconsistency.

Indirectness

No serious differences were found regarding the population, intervention and outcome within the studies included in the systematic review. The studies followed the PICO process. The evidence was therefore not downgraded for indirectness.

Imprecision

Imprecision was evaluated through the estimation of the optimal information size (OIS) criterion, as recommended in the GRADE Handbook. The criterion is that the total number of included patients in the systematic review should be greater than *the number of patients generated by a conventional sample size calculation for a single adequately powered trial.* If this is not the case, downgrading for imprecision should be considered. https://gdt.gradepro.org/app/handbook/handbook.html#h.qoxhi6qajv5t

https://www.cebm.net/wp-content/uploads/2014/12/Was-the-study-big-enough-Cafe-Rules.pdf

The relative risk reduction for febrile neutropenia with pegfilgrastim use has previously been estimated to 46 % on average in a systematic review. Likewise, the occurrence of febrile neutropenia has previously been estimated to somewhere between 13-21 % in a retrospective cohort (https://www.ncbi.nlm.nih.gov/pmc/articles/PMC6154917/). Based on these data, the occurrence of febrile neutropenia in a population treated with pegfilgrastim can be estimated to approximately 15% – ((0.46 x 0.15) x 100)) = 8.1 %. Thus, the event of febrile neutropenia in a control group is 15 % and 8 % in a pegfilgrastim-treated group. The sample size needed for OIS determination, based on the estimate of events in the control group when assuming $\alpha = 0.05$ and $\beta = 0.2$, was calculated as being 650 patients in total. The total cohort numbers for OS, PFS and AE estimation were 2.502, 2.277 and 2.380, respectively. The following samples size calculator was used for sample size estimation: https://clincalc.com/stats/samplesize.aspx.

Publication bias

Visually inspection of the corresponding funnel plot revealed asymmetry and Egger's test indicated asymmetry with a significant p-value (p = 0.0015). The evidence was thus downgraded by 1 for possible publication bias.

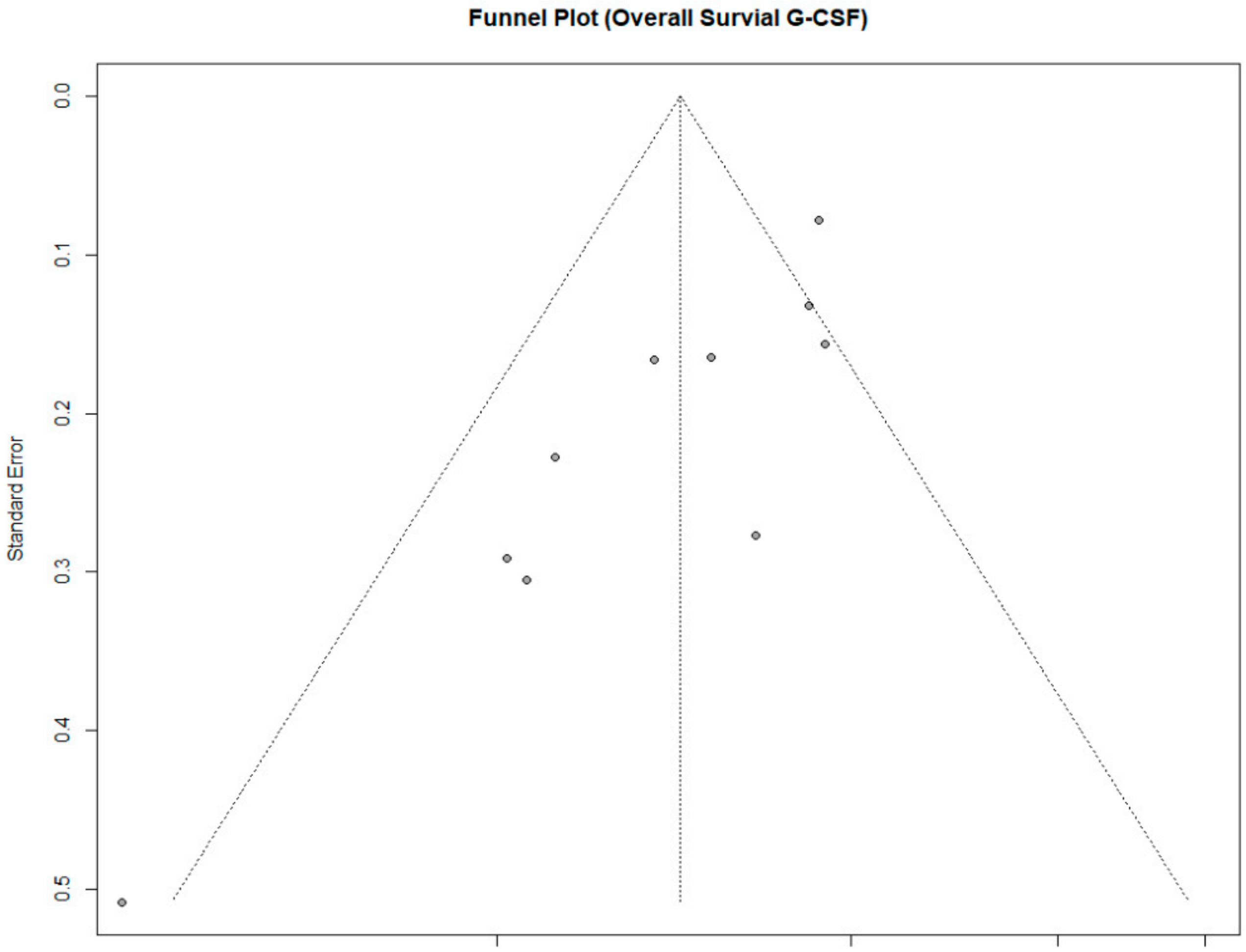

Factors which increase strength of evidence:

Large Effect

The HR estimate (0.72) did not reach large estimate definitions (< 0.5 or >2). Thus, the evidence could not be upgraded for large effect.

Dose–response gradient

Dose–response was not extracted from any of the included studies. The evidence was not upgraded for dose–response gradient.

Overall strength of the evidence

The overall strength of the evidence for OS and pegfilgrastim use was of low quality, as the evidence was downgraded by 1 for inconsistency of results and by 1 for possible publication bias. No other serious limitations were assessed.

PFS

Risk of bias

Risk of bias was assessed through the Newcastle–Ottawa Scale in retrospective cohort trials, and the Risk of Bias (RoB) tool was used on the RCTs. Overall, none of the included studies in the PFS and pegfilgrastim meta-analysis was of low quality.

Inconsistency of results

The heterogeneity of the results obtained from the time-to-event meta-analysis on PFS revealed a I^2 value of 73 %. The evidence was downgraded for inconsistency.

Indirectness

No serious differences were found regarding the population, intervention and outcome within the studies included in the systematic review. The studies followed the PICO process. The evidence was therefore not downgraded for indirectness.

Imprecision

Imprecision was evaluated through estimation of the optimal information size (OIS) criterion as recommended in the GRADE Handbook. The criterion is that the total number of included patients in the

systematic review should be greater than *the number of patients generated by a conventional sample size calculation for a single adequately powered trial.* If this is not the case, downgrading for imprecision should be considered. https://gdt.gradepro.org/app/handbook/handbook.html#h.qoxhi6qajv5t

https://www.cebm.net/wp-content/uploads/2014/12/Was-the-study-big-enough-Cafe-Rules.pdf

The relative risk reduction for febrile neutropenia with pegfilgrastim use has previously been estimated to 46 % on average in a systematic review. Likewise, the occurrence of febrile neutropenia has previously been estimated to somewhere between 13-21 % in a retrospective cohort (https://www.ncbi.nlm.nih.gov/pmc/articles/PMC6154917/). Based on these data, the occurrence of febrile neutropenia in a population treated with pegfilgrastim can be estimated to 15% – ((0.46 x 0.15) x 100)) = 8.1 %. Thus, the event of febrile neutropenia in a control group is 15 % and 8 % in a pegfilgrastim-treated group. The sample size needed for OIS determination, based on the estimate of events in the control group when assuming $\alpha = 0.05$ and $\beta = 0.2$, was calculated as being 650 patients in total. The total cohort numbers for OS, PFS and AE estimation were 2.502, 2.277 and 2.380, respectively. The following samples size calculator was used for sample size estimation: https://clincalc.com/stats/samplesize.aspx.

Publication bias

The visually inspection of the corresponding funnel plot revealed slight asymmetry, but Egger's test indicated no significant asymmetry (p-value = 0.1756). The evidence was thus not downgraded for possible publication bias.

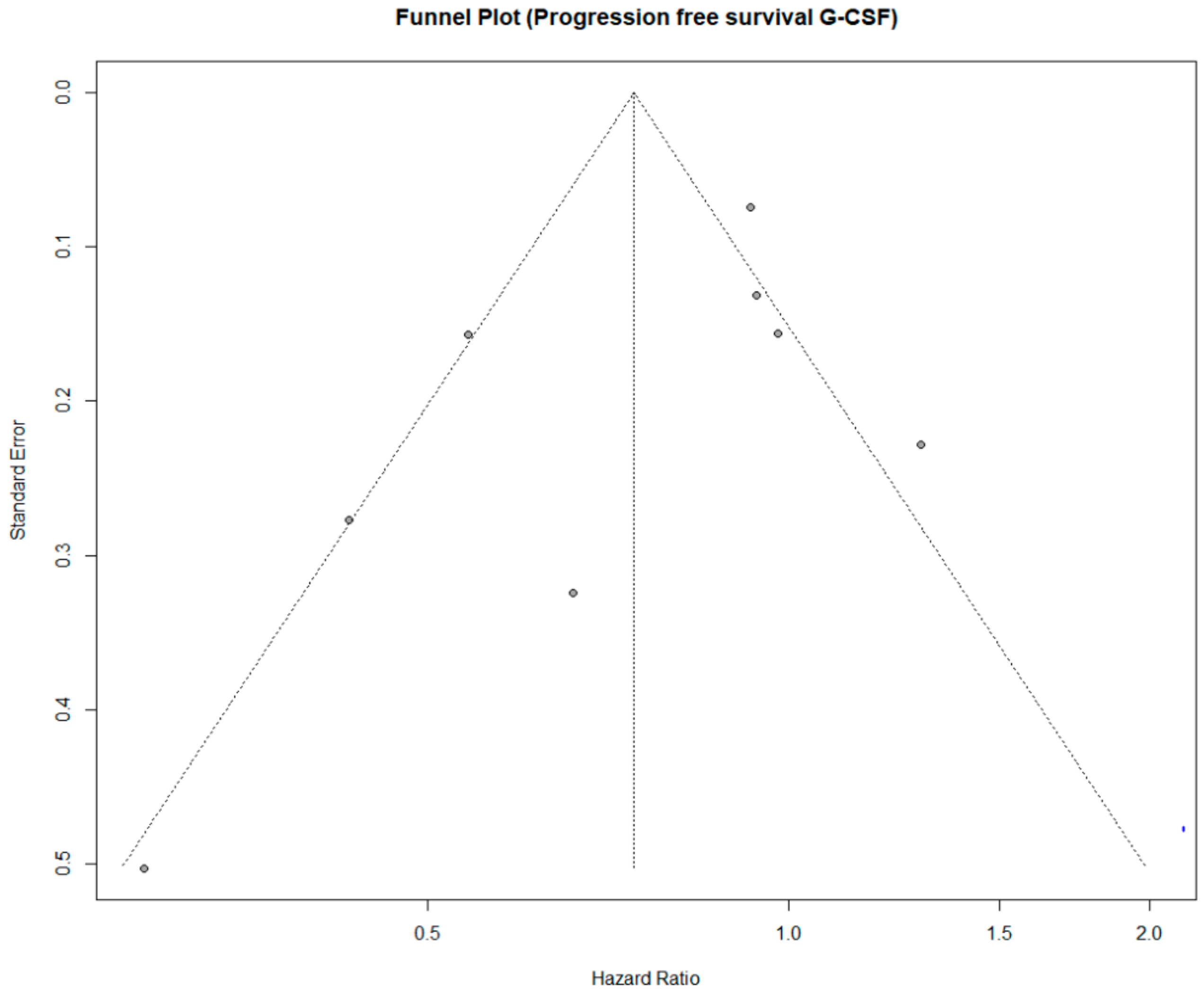

Factors which increase strength of evidence:

Large Effect

The HR estimate (0.74) did not reach large estimate definitions (< 0.5 or >2). Thus, the evidence could not be upgraded for large effect.

Dose–response gradient

Dose–response was not extracted from any of the included studies. The evidence was not upgraded for dose–response gradient.

Overall strength of the evidence

The overall strength of the evidence for PFS and pegfilgrastim use was of moderate quality, as the evidence was downgraded 1 for inconsistency of results. No other serious limitations were assessed.

AE

Risk of bias

Risk of bias was assessed through the Newcastle Ottowa Scale in retrospective cohort trials and the Risk of Bias (Rob) tool was used on the RCTs. Overall, none of the included studies in the AE and pegfilgrastim meta-analysis was of low quality. Thus, the evidence was not downgraded.

Inconsistency of results

The heterogeneity of the results obtained from the time-to-event meta-analysis on AE revealed a I^2 value of 72 %. The evidence was downgraded for inconsistency.

Indirectness

No serious differences were found regarding the population, intervention and outcome within the studies included in the systematic review. The studies followed the PICO process. The evidence was therefore not downgraded for indirectness.

Imprecision

Imprecision was evaluated through the estimation of the optimal information size (OIS) criterion, as recommended in the GRADE Handbook. The criterion is that the total number of included patients in the systematic review should be greater than *the number of patients generated by a conventional sample size calculation for a single adequately powered trial.* If this is not the case, downgrading for imprecision should be considered. https://gdt.gradepro.org/app/handbook/handbook.html#h.qoxhi6qajv5t

https://www.cebm.net/wp-content/uploads/2014/12/Was-the-study-big-enough-Cafe-Rules.pdf

The relative risk reduction for febrile neutropenia with pegfilgrastim use has previously been estimated to 46 % on average in a systematic review. Likewise, the occurrence of febrile neutropenia has previously been estimated to somewhere between 13-21 % in a retrospective cohort (https://www.ncbi.nlm.nih.gov/pmc/articles/PMC6154917/). Based on these data, the occurrence of febrile neutropenia in a population treated with pegfilgrastim can be estimated to 15% – ((0.46 x 0.15) x 100)) = 8.1 %. Thus, the event of febrile neutropenia in a control group is 15 % and 8 % in a pegfilgrastim-treated group.

The sample size needed for OIS determination, based on the estimate of events in the control group when assuming $\alpha = 0.05$ and $\beta = 0.2$, was calculated as being 650 patients in total. The total cohort numbers for OS, PFS and AE estimation were 2.502, 2.277 and 2.380, respectively. The following samples size calculator was used for sample size estimation: https://clincalc.com/stats/samplesize.aspx.

Publication bias

Visually inspection of the corresponding funnel plot revealed no serious asymmetry, and Egger's test indicated no significant asymmetry (p-value = 0.1635). The evidence was thus not downgraded for possible publication bias.

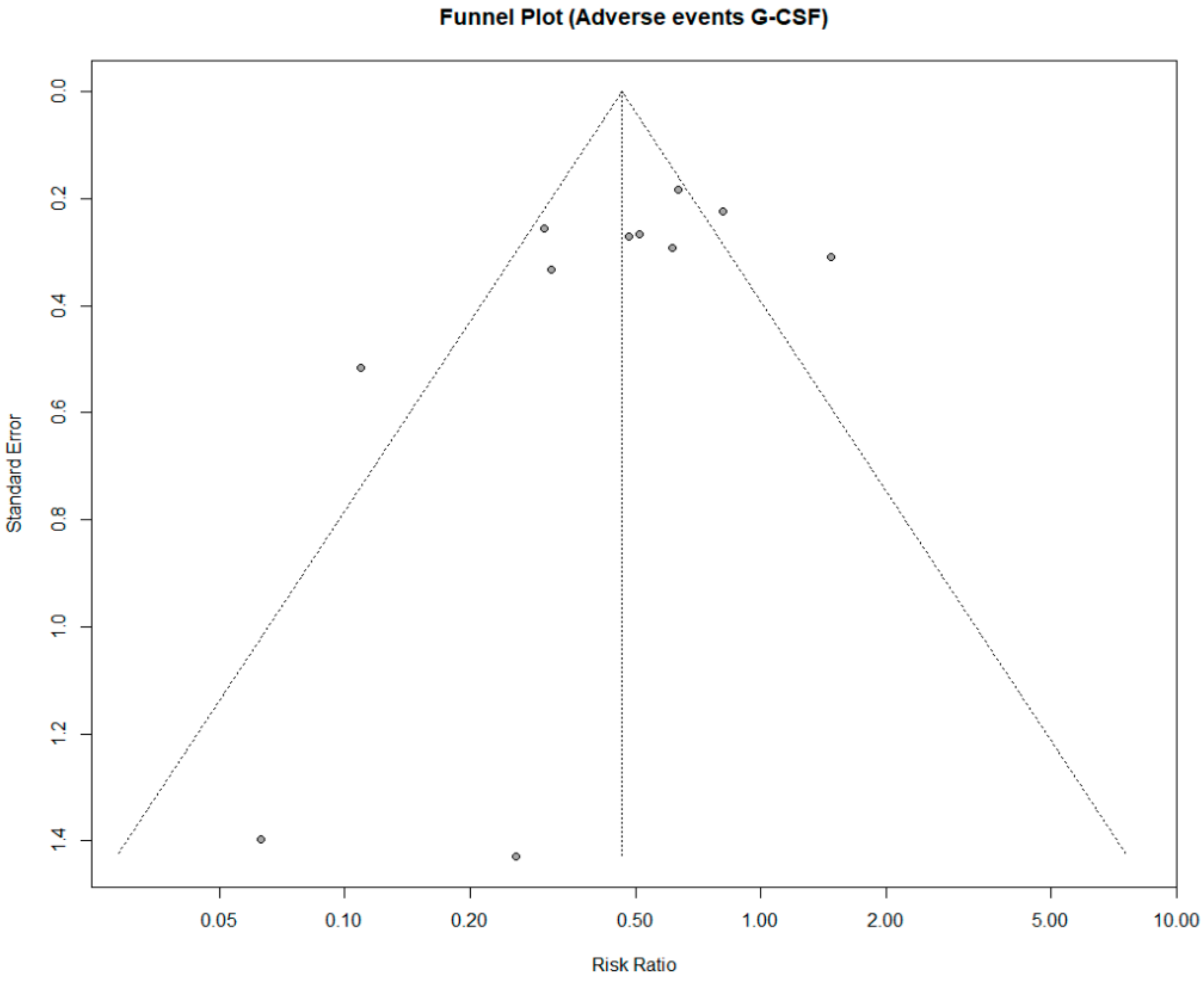

Factors which increase strength of evidence:

Large Effect

The RR estimate (0.46) reached large estimate definitions (< 0.5 or >2) and was significant (95 % CI: 0.28 – 0.77). Thus, the evidence was upgraded for a large effect.

Dose–response gradient

Dose–response was not extracted from any of the included studies. The evidence was not upgraded for dose–response gradient.

Overall strength of the evidence

The overall strength of the evidence for AE and pegfilgrastim use was of high quality. The evidence was downgraded by 1 for inconsistency of results but upgraded by 1 for a large and significant estimate (RR = 0.46, 95 % CI: 0.28 – 0.77). No other serious limitations were assessed.

Outcome	Number of studies	Study design / Limitations	Risk of bias	Inconsistency	Indirectness	Imprecision	Publication bias	Other considerations	HR/RR (95 % CI)	Overall Quality
**OS**	10	RCT + Cohorts / None	Not serious	Serious	Not serious	Not serious	Serious	None	HR = 0.72 (0.56-0.91)	⊕⊕◯◯ - Low
**PFS**	8	RCT + Cohorts / None	Not serious	Serious	Not serious	Not serious	Not serious	None	HR = 0.74 (0.51 – 1.08)	⊕⊕⊕◯ - Moderate
**AE**	8	RCT + Cohorts / None	Not serious	Serious	Not serious	Not serious	Not serious	Upgraded for large estimate	RR = 0.46 (0.28 – 0.77)	⊕⊕⊕⊕ - High

References

42. Rowinsky EK, Baker SD, Burks K, O'Reilly S, Donehower RC, Grochow LB. High-dose topotecan with granulocyte-colony stimulating factor in fluoropyrimidine-refractory colorectal cancer: a phase II and pharmacodynamic study. *Ann Oncol*. 1998;9(2):173-180. doi:10.1023/A:1008266630701
43. Saltz LB, Kemeny NE, Tong W, Harrison J, Berkery R, Kelsen DP. 9-Aminocamptothecin by 72-hour continuous intravenous infusion is inactive in the treatment of patients with 5-fluorouracil-refractory colorectal carcinoma. *Cancer*. 1997;80(9):1727-1732. doi:10.1002/(sici)1097-0142(19971101)80:9<1727::aid-cncr5>3.0.co;2-b
44. Ajani JA, Ilson DH, Daugherty K, Pazdur R, Lynch PM, Kelsen DP. Activity of taxol in patients with squamous cell carcinoma and adenocarcinoma of the esophagus. *J Natl Cancer Inst*. 1994;86(14):1086-1091. doi:10.1093/JNCI/86.14.1086
45. Donlon NE, Kammili A, Roopnarinesingh R, Davern M, Power R, King S, Chmelo J, Phillips AW, Donohoe CL, Ravi N, Lowery M, Mueller CL, Cools-Lartigue J, Ferri LE, Reynolds JV. FLOT-regimen Chemotherapy and Transthoracic en bloc Resection for Esophageal and Junctional Adenocarcinoma. *Ann Surg*. 2021;274(5):814-820. doi: 10.1097/SLA.0000000000005097.
46. Ohtsu A, Boku N, Muro K, Chin K, Muto M, Yoshida S, Satake M, Ishikura S, Ogino T, Miyata Y, Seki S, Kaneko K, Nakamura A. Definitive chemoradiotherapy for T4 and/or M1 lymph node squamous cell carcinoma of the esophagus. *J Clin Oncol.* 1999;17(9):2915-21. doi: 10.1200/JCO.1999.17.9.2915
47. Sharma A, Pramanik R, Kumar A, Pathy S, Kumar S, Bhoriwal S, Thulkar S, Dash NR, Pal S, Choudhary P, Pawar S, Kumar R, Gupta G. Safety and Efficacy of Modified FOLFIRINOX in Unresectable or Metastatic Gallbladder Cancer: A Phase II Pilot Study. *JCO Glob Oncol*. 2021;7:820-826. doi: 10.1200/GO.20.00657.
48. Catenacci DVT, Chase L, Lomnicki S, Karrison T, de Wilton Marsh R, Rampurwala MM, Narula S, Alpert L, Setia N, Xiao SY, Hart J, Siddiqui UD, Peterson B, Moore K, Kipping-Johnson K, Markevicius U, Gordon B, Allen K, Racette C, Maron SB, Liao CY, Polite BN, Kindler HL, Turaga K, Prachand VN, Roggin KK, Ferguson MK, Posner MC. Evaluation of the Association of Perioperative UGT1A1 Genotype-Dosed gFOLFIRINOX With Margin-Negative Resection Rates and Pathologic Response Grades Among Patients With Locally Advanced Gastroesophageal Adenocarcinoma: A Phase 2 Clinical Trial. *JAMA Netw Open*. 2020;3(2):e1921290. doi: 10.1001/jamanetworkopen.2019.21290.
49. Dalla Chiesa M, Tomasello G, Buti S, Rovere RK, Brighenti M, Lazzarelli S, Donati G, Passalacqua R. Sequential chemotherapy with dose-dense docetaxel, cisplatin, folinic acid and 5-fluorouracil (TCF-dd) followed by combination of oxaliplatin, folinic acid, 5-fluorouracil and irinotecan (COFFI) in metastatic gastric cancer: results of a phase II trial. *Cancer Chemother Pharmacol*. 2011;67(1):41-8. doi: 10.1007/s00280-010-1281-5.
50. Di Lauro L, Belli F, Arena MG, Carpano S, Paoletti G, Giannarelli D, Lopez M. Epirubicin, cisplatin and docetaxel combination therapy for metastatic gastric cancer. *Ann Oncol*. 2005;16(9):1498-502. doi: 10.1093/annonc/mdi281.
51. Felici A, Carlini P, Ruggeri EM, Gamucci T, Pollera CF, De Marco S, Fariello AM, Moscetti L, Gelibter A, Adami E, Sperduti I, Cognetti F. Bi-weekly chemotherapy with cisplatin, epirubicin, folinic acid and 5-fluororacil continuous infusion plus g-csf in advanced gastric cancer: a multicentric phase II study. *Cancer Chemother Pharmacol*. 2006;57(1):59-64. doi: 10.1007/s00280-005-0032-5.
52. Hejna M, Raderer M, Zacherl J, Ba-Ssalamah A, Püspök A, Schmidinger M, Pluschnig U, Brodowicz T, Zielinski CC. Phase II study of docetaxel in combination with oxaliplatin in patients with metastatic or locally advanced esophagogastric cancer previously untreated with chemotherapy for advanced disease: results of the Central European Cooperative Oncology Group Study ESGAS.1.2.001. *Anticancer Drugs*. 2008;19(5):535-9. doi: 10.1097/CAD.0b013e3282fb178a.
53. Makatsoris T, Papakostas P, Kalofonos HP, Xanthakis I, Tsavdaridis D, Aravantinos G, Gogas H, Klouvas G, Kosmidis P, Pectasides D, Fountzilas G. Intensive weekly chemotherapy with docetaxel, epirubicin and carboplatin with G-CSF support in patients with advanced gastric cancer: a Hellenic Cooperative Oncology Group (HeCOG) phase II study. *Med Oncol*. 2007;24(3):301-7. doi: 10.1007/s12032-007-0004-1.

54. Mavroudis D, Kourousis C, Androulakis N, Kalbakis K, Agelaki S, Kakolyris S, Souglakos J, Sarra E, Vardakis N, Hatzidaki D, Sarmonis G, Georgoulias V. Frontline treatment of advanced gastric cancer with docetaxel and granulocyte colony-stimulating factor (G-CSF): a phase II trial. *Am J Clin Oncol*. 2000;23(4):341-4. doi: 10.1097/00000421-200008000-00005.
55. Shin SJ, Jeung HC, Ahn JB, Rha SY, Yoo NC, Roh JK, Noh SH, Chung HC. Mobilized CD34+ cells as a biomarker candidate for the efficacy of combined maximal tolerance dose and continuous infusional chemotherapy and G-CSF surge in gastric cancer. *Cancer Lett*. 2008;270(2):269-76. doi: 10.1016/j.canlet.2008.05.011.
56. Bojic M, Pluschnig U, Zacherl J, Thallinger CM, Ba-Ssalamah A, Maresch J, Datler P, Schoppmann SF, Hejna M. Docetaxel, cisplatin and 5-fluorouracil plus granulocyte colony-stimulating factor prophylaxis in patients with metastatic adenocarcinoma of the stomach and gastroesophageal junction: experience at the Medical University of Vienna. *Anticancer Res*. 2011;31(6):2379-82.
57. Dirican A, Küçükzeybek Y, Tarhan MO, Somali I, Erten C, Demir L, Can A, Bayoglu IV, Akyol M, Ekinci N, Medeni M, Koyuncu B, Alacacioglu A. One-day DCF regimen in patients with metastatic gastric cancer. *Tumori*. 2013 Mar 1;99(2):145-148. doi: 10.1177/030089161309900204.
58. Liguigli W, Tomasello G, Toppo L, Poli R, Lazzarelli S, Negri F, Perrucci B, Curti A, Brighenti M, Donati G, Nazzari M, Martinotti M, Vismarra M, Rovatti M, Passalacqua R. Safety and efficacy of dose-dense chemotherapy with TCF regimen in elderly patients with locally advanced or metastatic gastric cancer. *Tumori*. 2017;103(1):93-100. doi: 10.5301/tj.5000556.
59. Ozdemir N, Abali H, Vural M, Yalcin S, Oksuzoglu B, Civelek B, Oguz D, Bostanci B, Yalcin B, Zengin N. Docetaxel, cisplatin, and fluorouracil combination in neoadjuvant setting in the treatment of locally advanced gastric adenocarcinoma: Phase II NEOTAX study. Cancer Chemother *Pharmacol*. 2014;74(6):1139-47. doi: 10.1007/s00280-014-2586-6. Epub 2014 Sep 19. Erratum in: Cancer Chemother Pharmacol. 2015 Jul;76(1):217. doi: 10.1007/s00280-015-2797-5.
60. Tomasello G, Dalla Chiesa M, Buti S, Brighenti M, Negri F, Kraft Rovere R, Martinotti M, Buononato M, Brunelli A, Lazzarelli S, Donati G, Passalacqua R. Dose-dense chemotherapy in metastatic gastric cancer with a modified docetaxel-cisplatin-5-fluorouracil regimen. *Tumori*. 2010;96(1):111-5. doi: 10.1177/030089161009600108.
61. Tomasello G, Liguigli W, Poli R, Lazzarelli S, Brighenti M, Negri F, Curti A, Martinotti M, Olivetti L, Rovatti M, Donati G, Passalacqua R. Efficacy and tolerability of chemotherapy with modified dose-dense TCF regimen (TCF-dd) in locally advanced or metastatic gastric cancer: final results of a phase II trial. *Gastric Cancer*. 2014;17(4):711-7. doi: 10.1007/s10120-013-0317-z.
62. Xiao J, Chen Y, Li W, Gong J, Zhou Z, Deng Y, Wang L, Ren D, Wang J, Peng J, Lan P. Dose-dense biweekly docetaxel combined with 5-fluorouracil as first-line treatment in advanced gastric cancer: a phase II trial. *Med Oncol*. 2015;32(2):334. doi: 10.1007/s12032-014-0334-8.
63. Tomasello G, Valeri N, Ghidini M, Smyth EC, Liguigli W, Toppo L, Mattioli R, Curti A, Hahne JC, Negri FM, Panni S, Ratti M, Lazzarelli S, Gerevini F, Colombi C, Panni A, Rovatti M, Treccani L, Martinotti M, Passalacqua R. First-line dose-dense chemotherapy with docetaxel, cisplatin, folinic acid and 5-fluorouracil (DCF) plus panitumumab in patients with locally advanced or metastatic cancer of the stomach or gastroesophageal junction: final results and biomarker analysis from an Italian oncology group for clinical research (GOIRC) phase II study. *Oncotarget*. 2017;8(67):111795-111806. doi: 10.18632/oncotarget.22909.
64. Li B, Yu J, Wang L, Li C, Zhou T, Zhai L, Xing L. Study of local three-dimensional conformal radiotherapy combined with transcatheter arterial chemoembolization for patients with stage III hepatocellular carcinoma. *Am J Clin Oncol.* 2003;26(4):e92-9. doi: 10.1097/01.COC.0000077936.97997.AB.
65. Bamias A, Syrigos K, Fountzilas G, Tzamakou E, Soulti K, Karavasilis V, Alamanos Y, Christodoulou C, Pavlidis N. Intensified bimonthly cisplatin with bolus 5-fluorouracil, continuous 5-fluorouracil and high-dose leucovorin (LV5FU2) in Patients with advanced gastrointestinal carcinomas: a phase I dose-finding and pharmacokinetic study. *Am J Clin Oncol*. 2004;27(5):465-71. doi: 10.1097/01.coc.0000128870.72525.c7.

66. Androulakis N, Kourousis C, Dimopoulos MA, Samelis G, Kakolyris S, Tsavaris N, Genatas K, Aravantinos G, Papadimitriou C, Karabekios S, Stathopoulos GP, Georgoulias V. Treatment of pancreatic cancer with docetaxel and granulocyte colony-stimulating factor: a multicenter phase II study. *J Clin Oncol*. 1999;17(6):1779-85. doi: 10.1200/JCO.1999.17.6.1779.
67. Napolitano F, Formisano L, Giardino A, Girelli R, Servetto A, Santaniello A, Foschini F, Marciano R, Mozzillo E, Carratù AC, Cascetta P, De Placido P, De Placido S, Bianco R. Neoadjuvant Treatment in Locally Advanced Pancreatic Cancer (LAPC) Patients with FOLFIRINOX or Gemcitabine NabPaclitaxel: A Single-Center Experience and a Literature Review. *Cancers (Basel).* 2019;11(7):981. doi: 10.3390/cancers11070981.
68. de W Marsh R, Talamonti MS, Baker MS, Posner M, Roggin K, Matthews J, Catenacci D, Kozloff M, Polite B, Britto M, Wang C, Kindler H. Primary systemic therapy in resectable pancreatic ductal adenocarcinoma using mFOLFIRINOX: A pilot study. *J Surg Oncol.* 2018;117(3):354-362. doi: 10.1002/jso.24872.
69. Sasaki M, Ueno H, Mitsunaga S, Ohba A, Hosoi H, Kobayashi S, Ueno M, Terazawa T, Goto M, Inoue D, Namiki S, Sakamoto Y, Kondo S, Morizane C, Ikeda M, Okusaka T. A phase II study of FOLFIRINOX with primary prophylactic pegfilgrastim for chemotherapy-naïve Japanese patients with metastatic pancreatic cancer. *Int J Clin Oncol.* 2021;26(11):2065-2072. doi: 10.1007/s10147-021-02001-y.
70. Scheithauer W, Kornek GV, Raderer M, Hejna M, Valencak J, Miholic J, Kovats E, Lang F, Funovics J, Bareck E, Depisch D. Phase II trial of gemcitabine, epirubicin and granulocyte colony-stimulating factor in patients with advanced pancreatic adenocarcinoma. *Br J Cancer*. 1999;80(11):1797-802. doi: 10.1038/sj.bjc.6690600.
71. Ghorani E, Wong HH, Hewitt C, Calder J, Corrie P, Basu B. Safety and Efficacy of Modified FOLFIRINOX for Advanced Pancreatic Adenocarcinoma: A UK Single-Centre Experience. *Oncology*. 2015;89(5):281-287. doi:10.1159/000439171
72. van der Sijde F, van Dam JL, Groot Koerkamp B, Haberkorn BCM, Homs MYV, Mathijssen D, Besselink MG, Wilmink JW, van Eijck CHJ. Treatment Response and Conditional Survival in Advanced Pancreatic Cancer Patients Treated with FOLFIRINOX: A Multicenter Cohort Study. *J Oncol.* 2022;2022:8549487. doi: 10.1155/2022/8549487.
73. Caron B, Reimund JM, Ben Abdelghani M, Sondag D, Noirclerc M, Duclos B, Kurtz JE, Nguimpi-Tambou M. Survival and Predictive Factors of Chemotherapy With FOLFIRINOX as First-Line Therapy in Metastatic Pancreatic Cancer: A Retrospective Multicentric Analysis. *Pancreas*. 2021;50(6):803-806. doi: 10.1097/MPA.0000000000001837.
74. Macdonald JS, Jacobson JL, Modiano M, Moore DF, Gandara DR, Schroder LE, Chapman RA. A phase II trial of etoposide, leucovorin, 5-FU, and interferon alpha 2b (ELFI) + G-CSF for patients with pancreatic adenocarcinoma: a Southwest Oncology Group study (SWOG 9413). *Invest New Drugs*. 2000;18(3):269-73. doi: 10.1023/a:1006486025196.
75. Mahaseth H, Brutcher E, Kauh J, Hawk N, Kim S, Chen Z, Kooby DA, Maithel SK, Landry J, El-Rayes BF. Modified FOLFIRINOX regimen with improved safety and maintained efficacy in pancreatic adenocarcinoma. *Pancreas*. 2013;42(8):1311-5. doi: 10.1097/MPA.0b013e31829e2006.
76. Stathopoulos GP, Mavroudis D, Tsavaris N, Kouroussis C, Aravantinos G, Agelaki S, Kakolyris S, Rigatos SK, Karabekios S, Georgoulias V. Treatment of pancreatic cancer with a combination of docetaxel, gemcitabine and granulocyte colony-stimulating factor: a phase II study of the Greek Cooperative Group for Pancreatic Cancer. *Ann Oncol*. 2001;12(12):1823-8. doi: 10.1023/a:1013925410275.
77. Combination Chemotherapy with Itraconazole for Treating Metastatic Pancreatic Cancer in the Second-line or Additional Setting - PubMed. Accessed June 15, 2023. https://pubmed.ncbi.nlm.nih.gov/26124377/
78. Stein SM, James ES, Deng Y, Cong X, Kortmansky JS, Li J, Staugaard C, Indukala D, Boustani AM, Patel V, Cha CH, Salem RR, Chang B, Hochster HS, Lacy J. Final analysis of a phase II study of modified FOLFIRINOX in locally advanced and metastatic pancreatic cancer. *Br J Cancer*. 2016;114(7):737-43. doi: 10.1038/bjc.2016.45.
